# Real-time estimation of horizontal gaze angle by saccade integration using in-ear electrooculography

**DOI:** 10.1371/journal.pone.0190420

**Published:** 2018-01-05

**Authors:** Ľuboš Hládek, Bernd Porr, W. Owen Brimijoin

**Affiliations:** 1 Medical Research Council/Chief Scientist Office Institute of Hearing Research - Scottish Section, Glasgow, United Kingdom; 2 School of Engineering, University of Glasgow, Glasgow, United Kingdom; Tokai University, JAPAN

## Abstract

The manuscript proposes and evaluates a real-time algorithm for estimating eye gaze angle based solely on single-channel electrooculography (EOG), which can be obtained directly from the ear canal using conductive ear moulds. In contrast to conventional high-pass filtering, we used an algorithm that calculates absolute eye gaze angle via statistical analysis of detected saccades. The estimated eye positions of the new algorithm were still noisy. However, the performance in terms of Pearson product-moment correlation coefficients was significantly better than the conventional approach in some instances. The results suggest that in-ear EOG signals captured with conductive ear moulds could serve as a basis for light-weight and portable horizontal eye gaze angle estimation suitable for a broad range of applications. For instance, for hearing aids to steer the directivity of microphones in the direction of the user’s eye gaze.

## Introduction

Following a conversation in noisy environments can be challenging for hearing aid users because hearing aids amplify noise together with the target signal. Thus, hearing aids are often equipped with directional microphones, which attenuate background noise and amplify only the signals originating in front of the listener. In a typical conversation, however, the conversational partners can be outside the amplification pattern and the hearing impaired people adopt a strategy to follow a talker with the eyes [1–3]. The hearing devices do not take into account the eye movements; and therefore, it would be desirable that hearing prostheses were able to adapt according to the direction of eye gaze. Some authors have suggested that using eye gaze angle to steer hearing aid directional microphones could be of benefit to a listener [4,5]. How eye gaze is measured, however, remains an open question. The most reliable methods for mobile eye tracking involve cameras mounted on glass frames, but the cameras obstruct the field of view [6], and not every hearing aid user is willing to wear glasses.

A viable candidate for measuring eye gaze angle is electrooculography (EOG) which measures an electrical signal that arises from the rotation of electrically charged eyeballs. Consequently, electrodes placed in the vicinity of the eyes can measure these potentials, and the magnitude of these potentials depend on the eye gaze angle. EOG has many practical applications including wheelchair control [7,8], activity recognition [9,10], retinal function testing [11,12], sleep stage classification [13], or as a general gaze control interface [14–16]. It is also known as an artifact of electroencephalography (EEG) [17]. However, its full potential for hearing aids (or indeed any mobile applications) has not been fully recognized, mainly because the EOG is typically measured by using large obtrusive electrodes that are attached to the sides of the head. Due to the electrical properties of the body, however, EOG can be measured anywhere on the head, although its magnitude varies with the electrode placement. When measured in peri-orbital positions it usually has values 8–33 μV/ 1° of visual angle, and around 3 μV /1° can be measured inside the ear canals [11,18]. This finding suggests that eye movements can be analysed by hearing aids with nothing more than conductive ear-moulds.

Eye movements are seen in the EOG signal as a change of the potential across two electrodes placed either horizontally or vertically around the eyeballs. The analysis of EOG is usually based on detection of saccades, fixations, and blinks [10,14–16,19–21]. Saccades are the most common type of eye movement, and they are characterized by a rapid change of the eye position between two relatively stable fixation points. They produce very distinct patterns in the EOG voltage, which are relatively easy to detect because the deflections have magnitudes which are above the usual high-frequency noise level, and they are short in duration. Microsaccades are tremor-like movements during fixation periods but they produce relatively small EOG signals that are difficult to detect. Other types of eye movements such as smooth pursuit, vestibulo-ocular reflex, vergence movement, nystagmus, or optokinetic reflex could be analysed by EOG, but they are not in the focus of this study.

Saccade detection algorithms often claim near perfect detection rates. However, the performance of these algorithms vary with the quality of the EOG recording, which is influenced by electrode types, the electrode placements, lighting conditions [11], and the degree of physical activity. Most methods are based on the analysis of the derivative of the EOG signal and subsequent classification. The derivative function can be understood as a high-pass (HP) filter with a cut-off frequency proportional to the sampling rate. The output of the derivative is usually very noisy, and therefore various approaches proposed ways to increase the signal to noise ratio. The method [14] used a rule-based algorithm to classify the derivative output as a saccade if the derivative changed the sign. The methods [15,20] employed probabilistic feature-based classification using Gaussian mixture models on the derivative output. The methods [10] and [16], instead of the derivative, analysed parameters of continuous wavelet transformation. The transformation parameters are then used as an input into a neural network or nearest neighbour classifier. Yet another method [19] analysed the second derivate (acceleration) of the EOG signal. The saccades were then detected by thresholding the acceleration values, and the threshold was adapted based on the previous measurements. Although these methods perform well, they have not considered the saccade magnitude as a predictor and using this predictor can possibly improve the performance of the detection algorithm.

Obtaining the saccade magnitude from EOG will, however, require a calibration of the EOG signal to the eye gaze angle [16]. Under ideal conditions, the relationship is straightforward: EOG = constant * sin(eye angle) for all eccentricities, and this linear relationship holds for small and intermediate eccentricities. However, the actual relationship depends on the placement of electrodes, properties of body tissue, the shape of the head and other factors.

Various techniques detect saccades, blinks, and fixations, but only a few estimate the actual eye gaze angle from EOG. For instance, the method [22] estimated eye gaze using a comparison of EOG signals from multiple electrodes in different locations around the eyes. The method took into account the non-linear relationship of the signals from different measurements sites, which enabled it to cancel out the errors. The method reported the accuracy of about 4°. Such an approach, however, is inapplicable to a setting with only one EOG channel. In another work [23], an external video was used to calibrate the system. The method was based on a comparison of the saliency maps [24] and the EOG signal. This technique achieved an estimation error of about 15°. However, this method is also not suitable for applications without an external video source. In summary, the state-of-the-art technology [6] does not provide a solution to estimate the actual gaze position from a single-channel recording. The reason is that EOG has previously been considered for the detection of only relative—not absolute—changes of eye position. In this paper, we aim to challenge this view by assuming that eye position can be restored by integrating past saccades. Such an approach will lead to noisy predictions, but we argue that the level of noise will be acceptable for some applications.

**Fig 1:**
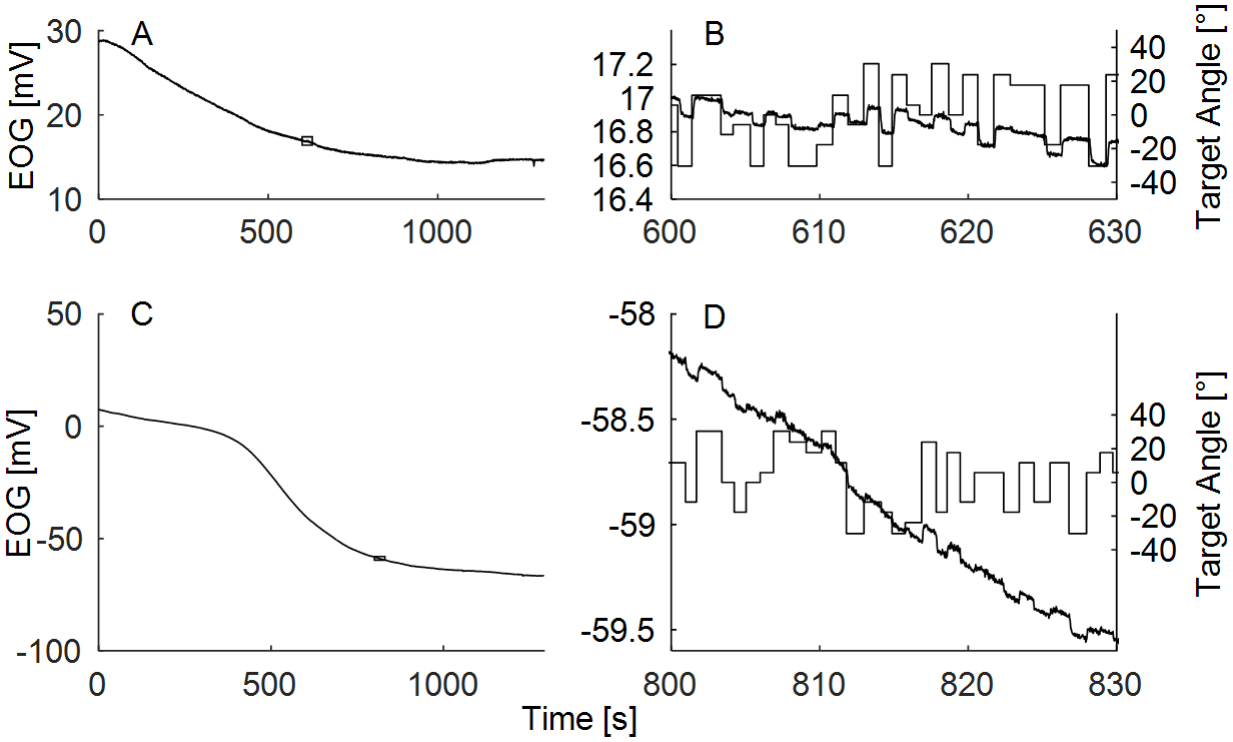
Raw in-ear EOG. (A,C) Two samples of 22-minute recordings of raw in-ear EOG. (B,D) Detailed view of the EOG waveform on a scale of 20 seconds. Straight solid lines denote the position of the visual targets. Small rectangles in the panels A and C indicate where the detailed views were taken from. The scale of y-axis in the zoomed in-panels B and D was kept fixed to 2 mV, x-axis was fixed to 20 s, and it shows actual time during the experiment.

The EOG signal is often polluted by various sources of noise [12], which are usually difficult to eliminate by simple filtering. The most dominant noise component in the EOG is the direct current (DC) drift. The drift can be characterized as a low-frequency noise (less than 1 Hz) with unstable spectral structure and with magnitudes up to several hundred mV. DC drift is inherent to any process involving electrodes attached to the skin [25], and it arises from the imbalance of the half-cell potentials of the two electrodes. When skin conductance changes (e.g., when sweat is released), the salt concentrations of the electrode gels change, and any differences between the two electrodes result in a slowly changing DC potential. The voltage changes that correspond to the actual EOG are small, and they ride on top of this large DC component (Fig 1). Although it is theoretically possible to decrease the drift in laboratory environments [12], this is not expected in real settings. The second largest source of noise is muscle activity [10]. These artifacts are stronger if the electrodes are placed closer to the muscles generating the electrical activity such as eye muscles, facial muscles, jaw muscles, neck muscles, tongue, or limbs. For example, vertical EOG electrodes pick up eye blinks whereas horizontal electrodes are less affected or not affected at all [21]. These artifacts are present in any type of muscle activity, and they can be seen as a broadband noise with magnitudes similar or greater than EOG. Electrical activity of the brain itself has virtually no impact on the EOG because of its very low amplitudes in comparison to the EOG signal. The synchronized activity of specific brain regions could potentially influence EOG, but again only at very low amplitudes and not at the locations where EOG is recorded. Finally, noise may arise from external sources including environmental and powerline noise which are picked up by the wires of the measurement device. These are typically limited to a narrow spectral region (e.g., 50 Hz or 60 Hz) and can easily be overcome by notch filters. Although the noise negatively impacts the quality of the EOG signal, transient events, such as saccades, are less error-prone to external noise due to their transitory nature. Saccade detection is therefore relatively reliable, even if the signal is corrupted.

The primary aim of this manuscript is to determine whether eye gaze direction can be estimated from the in-ear EOG recordings. Specifically, we aim to test a saccade integration algorithm and compare it to the output of the high-pass filtering approach. The saccade integration algorithm is a novel approach to estimate the actual eye gaze angle. It relies on the assumption that the variance of the eye gaze direction can be explained mainly by the saccadic movement, and only to a small extent by other types of eye movements. In this work, the saccade integration scheme has the following assumptions: 1) every eye movement can be characterized as an instantaneous step-like change of location, and all other types of eye movements can be ignored, 2) the eye is perfectly still during the fixation period, 3) there exists an approximately linear relationship on a short time scale between the change of the EOG signal and the change of the eye position [16], 4) noise related to the estimation of saccade magnitude has a normal distribution, 5) eye gaze is constrained by physical limitations, and 6) the head remains still. In future, the sixth assumption could be omitted, and the information about head movements could be used to enhance the estimation of eye gaze angle [26]. However, in this initial work, we decided to keep the head fixed. The model prediction is that the performance of the integration scheme will be better than working directly on the HP-filtered EOG.

For the remainder of the paper, we define these two approaches as:

- EOGHP, where the HP-filtered EOG is directly used for eye gaze angle estimation;
- SACCINT, where the HP-filtered EOG is fed into a saccade detector, and then the result is integrated.

This manuscript describes the eye gaze estimation scheme using in-ear EOG recordings and compares the output to the actual eye position monitored by a video-based eye tracker.

## Methods

### Participants

Seven normal- or corrected-to-normal-sighted human participants participated in the experiment. One participant could not perform the task with the eye tracker, and one participant was equipped with different type of electrodes. The data of the five remaining participants were used in the subsequent analysis. This study was approved by the West of Scotland Research Ethics Service. The participants were members of the Institute of Hearing Research, and they provided written informed consent.

### Setup and procedures

**Fig 2:**
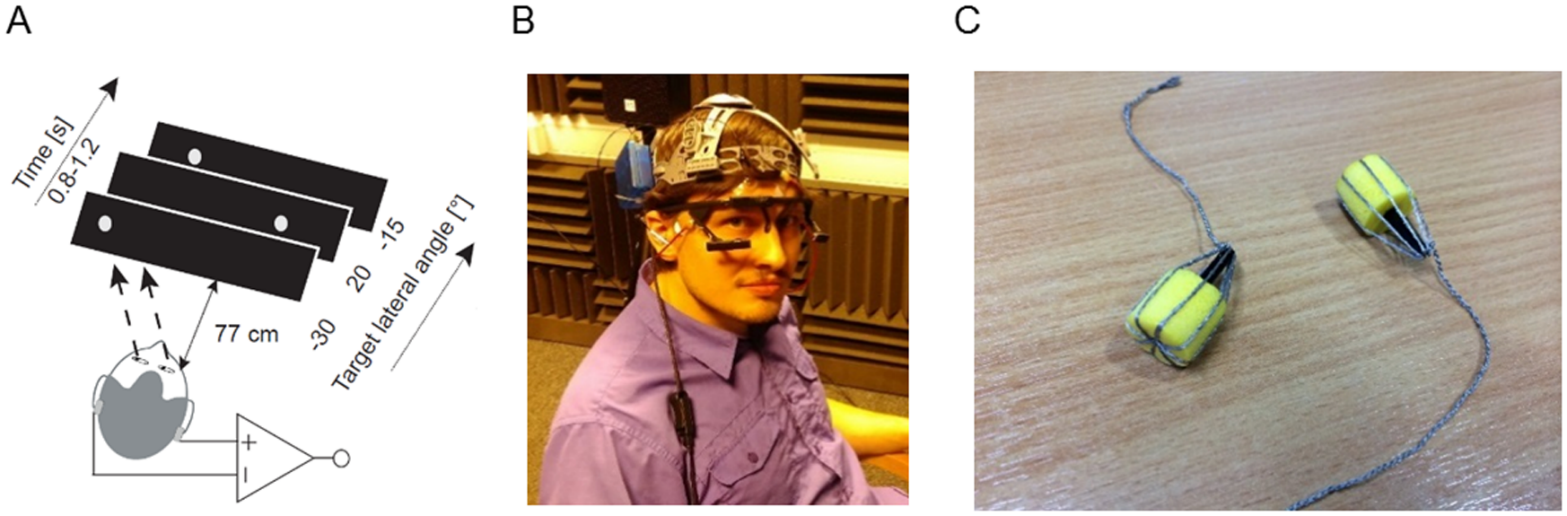
Experimental apparatus. (A) Schematics of the experimental procedure. (B) The participant was equipped with the mobile bio-amplifier (blue box) attached to the headband and the video-based eye tracker. (C) Detail of the in-ear electrodes.

The experiment was conducted in a testing booth (4.6 m x 4.1 m x 2.5 m—l x w x h) with lights turned off during the experiment. The acoustically treated room is one of the booths which are commonly used for auditory experiments. The participants were seated directly in the front of a 40” LCD screen (Samsung, UE40ES5500) at distance of 77 cm from the screen to the eyes (Fig 2A). The participants’ heads were not restrained or supported, but the participants were instructed to remain still and fixate on a small white dot (1° of visual angle) on a grey background at the height of their eyes. The position of the dot was drawn from a pseudorandom de Bruijn sequence [27] of 11 possible target locations that spanned from the left to right margins of the screen, covering approximately ± 30.5° of visual field. The dots changed their position after a pseudorandom interval of 0.8–1.2 s. The sequence was constructed so that all positions were equally represented and the transition between each possible pair of the positions occurred exactly 11 times, which led to 11^3^ (1331) total target presentations. The actual measurement period lasted about 22 minutes (excluding preparation). In the offline analysis, the measurement was split into a training period, in which the parameters of the model were estimated, and a testing period, in which the performance of the system was assessed.

A DC-coupled differential bio-amplifier (Attys, Glasgow Neuro LTD, UK) and a video-based eye tracker (Pupil Labs, Berlin, Germany) [28] that served as ground truth were used for the recording (Fig 2B). The bio-amplifier was equipped with a low-noise 24-bit sigma-delta AD converter and a Bluetooth transmitter which transmitted the measurements to the experimental computer at a sampling frequency (fs) of 83.34 Hz. When a measurement was unavailable, the previous value was used instead. Two disposable conductive ear moulds (Fig 2C) were made of ER1-14A ear tips (Etymotic Research, Elk Grove Village, IL, USA) and conductive thread (Electro Fashion, Kitronic, Nottingham, UK). The electrodes were attached to the bio-amplifier by 20 cm long non-shielded cables, and a small portion of electrode gel was put on the tip of the electrodes before insertion into the ear canal. The ground electrode was connected to the forehead with a regular medical grade Ag-Cl electrode. The bio-amplifier was held near the head using an adjustable plastic headband sourced from the inside of a construction hard hat. The ground truth eye tracker was connected to a dedicated Linux computer running eye tracker software (Pupil Capture, v0.7.5). The data from the ground truth eye tracker were collected for both eyes at 60 Hz with a resolution of 800 x 600 pixels. The eye tracker software directly outputted the eye gaze angle using a 3D eye model. The eye tracker computer transmitted the measurements via the local network to the experimental computer, where all recordings were kept for offline analysis. The experimental computer executed custom Matlab (v8.6.0, Natick, USA), Psychtoolbox [29–31] and Python (v3.5.1) scripts, which controlled the pace of the experiment.

### Ground truth

The eye data outputted by the eye tracker software were calibrated to the actual positions using histograms of all measurements. Subsequently, a linear transformation was applied to match the measurements with the positions of the targets. The resulting eye gaze angle was computed as a mean of the angles from the left and right eyes. The eye tracker and the bio-amplifier data were then temporally aligned using timestamps recorded by the eye tracker and the timestamps obtained by the experimental computer during the recording of the bio-amplifier data. For the purpose of the analysis, saccades from the ground truth were detected using the EyeMMV toolbox [32] with the following settings: minimum saccade duration was set to 50 ms, spatial parameters were set to x = 0.06 and y = 0.05 of the tracker units (normalized to 0–1) using the option ‘3s’ (i.e., the fixation cluster is defined as three standard deviations from the centre). In order to evaluate the system, the saccades obtained from the eye tracker were matched to the saccades estimated from the EOG data. Two saccades were matched if they were temporally closest to each other (sooner or later) and if

$$\left|{sacc}_{EOG}\right|<\left|{1.5\times sacc}_{GT}\right|+10$$ (1)

where |sacc_EOG_| is the magnitude of the EOG saccade and |sacc_GT_| is the magnitude of the saccade obtained from the ground truth. That means that the computed magnitude of the saccade had to be less than 1.5 times the magnitude of the ground truth saccade plus 10° (i.e., only the saccades of approximately equal magnitudes could be matched). These corrections were used to ensure correct matching when the EOG and the ground truth signals were not perfectly aligned in time, which was a side effect of the wireless transmission. In order to minimize the problems related to the delays and to make sure that the matching procedure worked as expected, matching was visually checked. We concluded that the delays between the EOG and the eye tracker had only a small impact and the delay could not influence the difference between the methods which are compared in this manuscript.

### Saccade integration scheme

**Fig 3:**
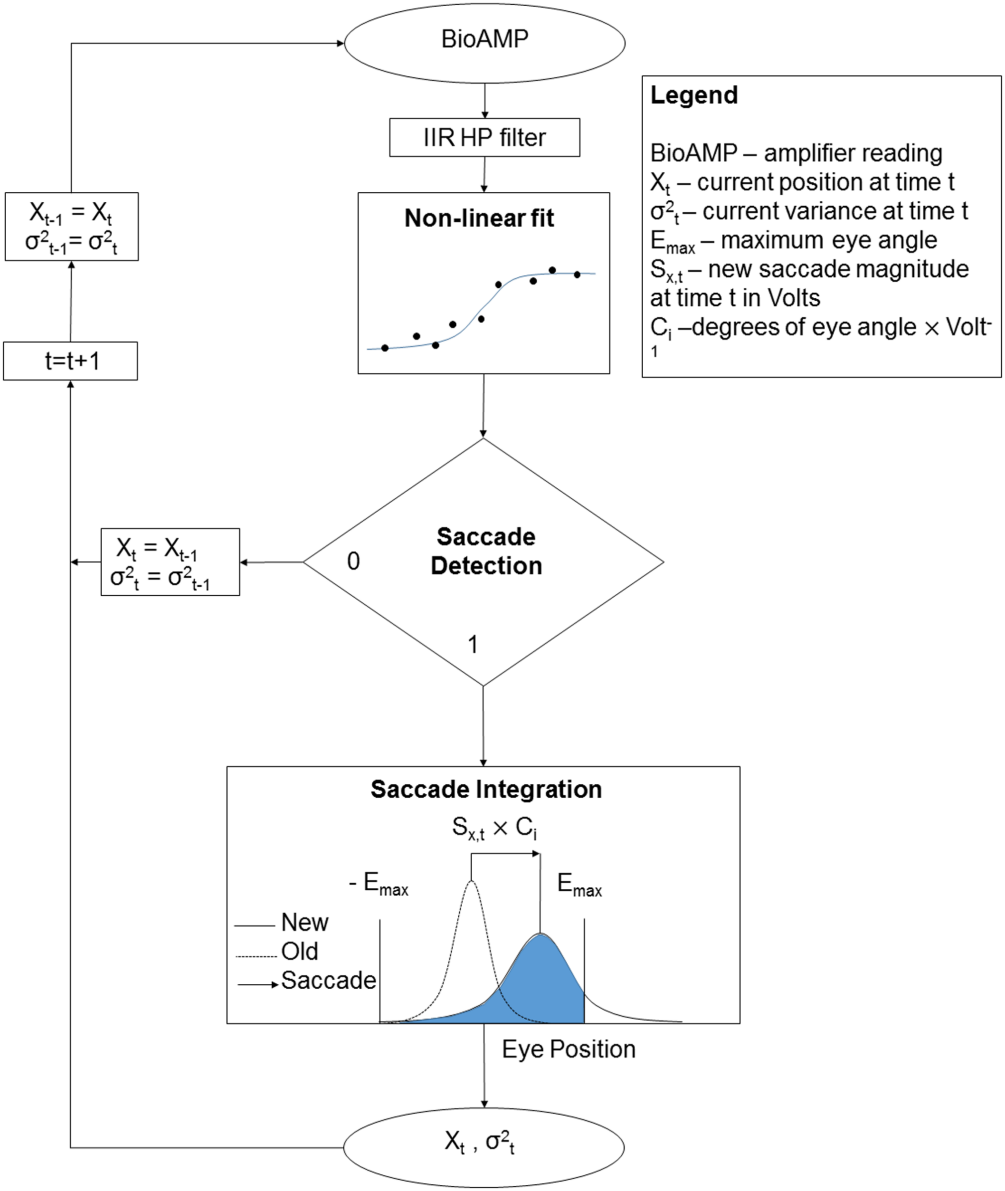
Model scheme.

The proposed EOG to eye gaze algorithm, SACCINT (Fig 3), used a single-channel EOG signal as an input. Ground truth measurements served to calibrate the system before the actual testing. The algorithm outputted eye gaze angle with a theoretical delay of up to 200 ms, which corresponded to half of the temporal window (see below) and the delay due to the HP filter. The analysis was run offline, though the algorithm can be run in real-time. In the first step of the scheme, the HP-filtered signal of the length of the temporal window was used to estimate three parameters of a non-linear model of a saccade, which was an s-shaped function: magnitude S_x_, gain S_g_, and temporal offset S_o_. These parameters were evaluated to confirm the saccade detection, and they were then used in subsequent integration. The saccades were identified when the time offset parameter S_o_ crossed the midline of the temporal window and when the magnitude of the signal deflection S_x_ was in a range defined by a minimum (N_x,min_) and a maximum of 70°. In the integration step, the saccades were represented as noisy measurements using a Gaussian probability density function (PDF). Subsequently, they were integrated over time. The integration had two steps. First, the mean of a new PDF was obtained as a sum of the previous mean and the magnitude of the new saccade. The variance was increased by a constant N_m_ which represented noise related to the measurement. In the second step, the PDF was clipped at the fixed boundaries ±EA_nax_. The algorithm used one more parameter C_i_ [°/mV], which defined the linear relationship between the change of EOG and the change of the horizontal eye gaze angle. C_i_ was estimated for each participant (index i). The parameters C_i_, N_m_, and N_x,min_ were calibrated for the whole dataset during the training phase using the ground truth measurements.

### High-pass filtering

The bio-amplifier readings were HP filtered with a second-order Butterworth filter with the cut-off frequency f_HP_. The purpose of the filtering was to remove as much of the low-frequency noise as possible but at the same time preserve information regarding eye position.

**Fig 4:**
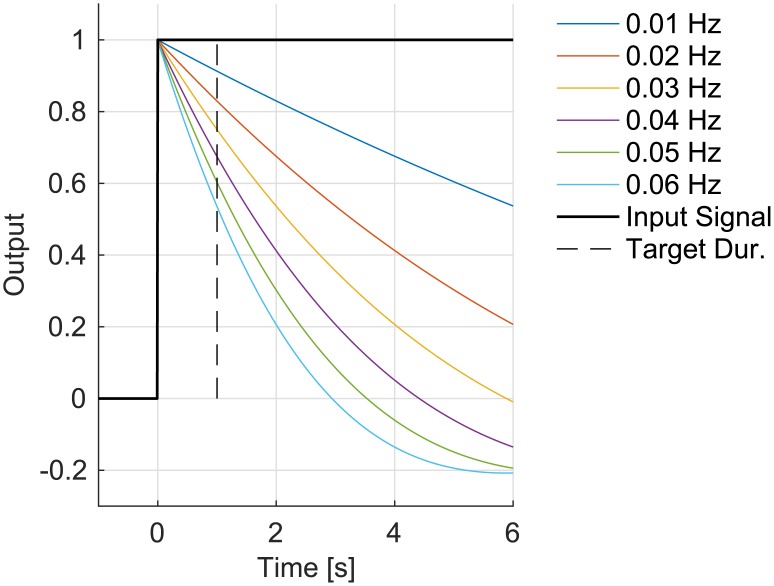
Step response of the IIR HP Butterworth filters as a function of half-power frequency.

Fig 4 shows the step response of HP filters with cut-off frequencies between 0.01 and 0.06 Hz for up to 6 seconds (6 seconds is a reasonable time for a very long off-axis fixation). The HP filter with cut-off frequency of 0.01 Hz reduces the step signal only by ~9% after 1 second (which was the typical duration of the visual target in this experiment) and it does not cross 0 even after 6 seconds. On the other hand, the HP filter with 0.06 Hz cut-off reduces the signal by ~47% after 1 second and crosses zero at less than 3 seconds. While the first example would affect a typical eye movement to a small extent, the latter example would change the slope—particularly for long fixations.

At this HP filtering stage of the algorithm, the traditional EOGHP algorithm took this signal and calculated the eye angle directly. The SACCINT algorithm, however, further processed the HP-filtered signal as described in the following section.

### Non-linear fit

The next step of the SACCINT algorithm was to fit the non-linear function to the HP-filtered signal. The signal was used to estimate the parameters of the simple saccadic model, which consisted of the s-shaped function:

$${f(u)=S}_{x}\text{tanh}(S_{g}(u+S_{o}))-AVG(S_{x}\text{tanh}\left(S_{g}(u+S_{o})\right))$$ (2)

The fit was obtained using a standard non-linear fitting procedure with constraints [33] with a limit of 20 iterations, and the objective function defined as the least square error. The parameters were constrained with the following values: So = <-136 ms, 136 ms>, Sg = <-150,150>, Sx = (0 mV, 1,05 x (max(x_EOG_)-min(x_EOG_))) /2 mV> where x_EOG_ is the EOG signal in the temporal window. The initial estimates for the three parameters were obtained as a weighted average of the previously estimated value and a pseudo randomly selected value from the above intervals with weights 0.95 and 0.05, respectively. The fit was repeated until the root mean square (RMS) error of the fit was below 4/C_i_ with a maximum of three repetitions but with completely random initial starting points within the accepted range of the constraints. The length of the temporal window was set 273 ms (23 measurements), which covers the duration of a typical saccade.

### Saccade detection

**Fig 5:**
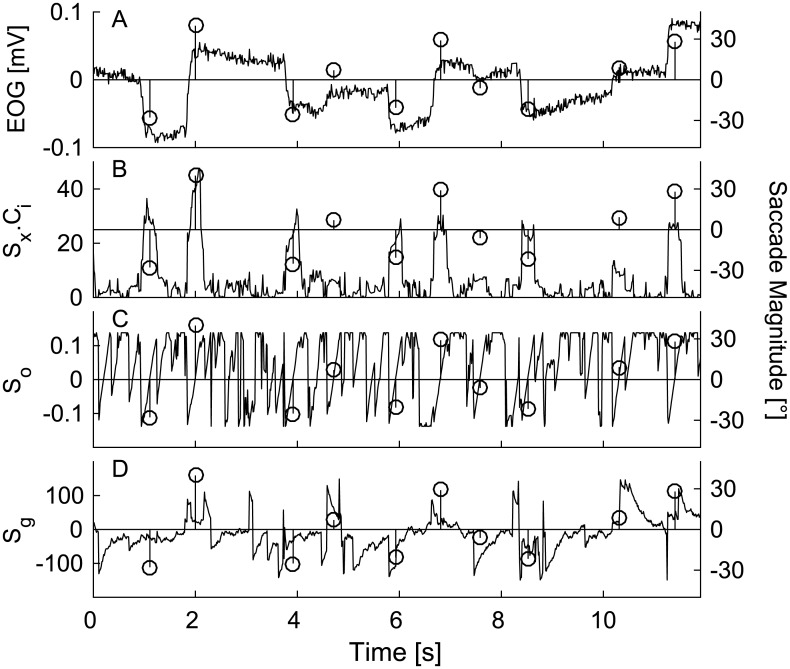
Detail of the saccade detection algorithm over 12 seconds of the experiment. An example of EOG recording (A) and (B)-(D) estimated parameters S_x_, S_o_, and S_g_. (B) S_x_ saccade magnitude parameter. (C) S_o_—saccade time shift parameter. (D) S_g_—saccade gain parameter. Circles with lines show detected saccades and their magnitude (right y-axis). The magnitude of a saccade was obtained by multiplying measured voltage S_x_ with C_i_ parameter obtained in the training period.

Fig 5A shows 12 seconds of EOG recording together with derived parameters (Fig 5B) S_x_ x C_i_ (Fig 5C) S_o_ (Fig 5D) S_g_ on the left y-axis. The most important parameter for saccade detection was S_o_. As a deflection in the EOG appeared in the temporal window, S_o_ progressively increased from negative values and changed its sign to positive when the deflection was in the middle of the window [14]. The second identifier of a saccade was its magnitude S_x_ due to physiological limits of eye. Thus the largest accepted saccade was N_x,max_, which was set to 70°. The smallest accepted saccade (N_x,min_) was a parameter likely to affect the number of detected saccades. Therefore, the influence of this parameter was investigated as a part of the study. The input for the detection algorithm were the parameters estimated at the time t and t-1.

Following a set of rules was used for saccade detection in each time t.

$$S_{x,t}\times C_{i}\in \;\langle N_{x,min},N_{x,max}\rangle$$ (3)

$$S_{x,t-1}\times C_{i}\in \;\langle N_{x,min},N_{x,max}\rangle$$ (4)

$$S_{o,t}\in (0,4/fs\rangle$$ (5)

$$S_{o,t-1}<\langle -4/fs,0\rangle$$ (6)

The first and second conditions [(3) and (4)] define the boundaries for saccade magnitude in the current estimate (at time t), and the estimate from the previous step (at time t-1). The third and fourth conditions [(5) and (6)] define the zero-crossing time of parameter So between t and t-1, and limit that time to be no farther than 4/fs from zero. Although it would be possible to use more conditions and achieve better detection performance, or use a statistically based model; here we aimed to demonstrate that a simple rule-based model is capable of saccade detection, and restoring actual eye gaze angle.

### Saccade integration

Saccade integration is a novel method of estimating eye gaze angle. It is based on an assumption that in many real situations eye gaze behaviour can be characterized solely in terms of saccades and fixations. It relies on the fact that eye positions resemble a normal distribution with the mean in the midline of the visual field [34], and that eye positions are naturally limited. The scheme is based on the step-like saccades (i.e. step changes) and stable fixations (i.e., no movement). A simple summation of the saccades would be unstable because (a) the estimation of saccade magnitude is noisy, (b) very small saccades cannot be detected, (c) the eye is not stable during the fixation period, and (d) any detection algorithm on a noisy signal will always have false alarms and misses. As a result, a simple summation of noisy estimates of saccades would lead to integration errors (e.g., the estimates could depart from the natural boundaries).

One way of mitigating these problems is to represent the eye position as a Gaussian PDF with the mean X_t,i_ and variance σ^2^_t,i_. If a saccade is detected, the magnitude of the new saccade in degrees is added to the mean of the previous estimate.

$$X_{t,i}^{'}=X_{t-1,i}+{sacc}_{EOG,t,i}$$ (7)

where X_t-1,i_ is the previous estimate. The magnitude of saccade at time t is defined as

$${sacc}_{EOG,t,i}=sign(S_{g,t}){\times S}_{x,t}\times C_{i}$$ (8)

and the variance of the PDF increases by the measurement noise N_m_:

$$\sigma_{t,i}^{'2}=\sigma_{t-1,i}^{2}+N_{m}^{2}$$ (9)

The new estimates of position (X_t,i_) and variance (σ^2^_t,i_) are computed by simulation of the newly obtained PDF, which is a truncated Gaussian distribution with the mean X’_t,i_ and variance σ’^2^_t,i_ clipped at the constraints *EA*_*max*_ = ±35°.

In order to characterize the saccade integration scheme, let us assume a perfectly performing saccade detector. In our case, this was obtained from the video-based eye tracker. Hence, there are two parameters N_m_ and N_x,min_ which influence the performance of the saccade integration.

**Fig 6:**
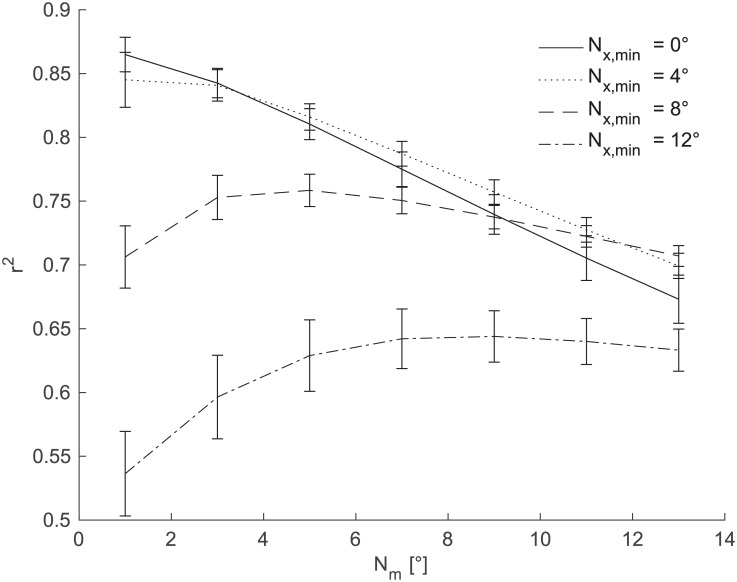
Limits of the saccade integration scheme. The figure shows the performance of the saccade integration scheme for the current experiment assuming the perfect saccade detection. The r^2^ values of the estimated and actual eye gaze position (y-axis) were computed for different values of N_m_ and N_x,min_. The N_m_ is on the x-axis, N_x,min_ is represented by different types of line. The lines show across-subject means; error bars show standard errors of the mean (SEM).

Fig 6 shows the effect of the N_m_ and N_x,min_ parameters on the performance of the saccade integration scheme, with N_m_ on the x-axis and different line styles showing different values of the N_x,min_ parameter. The performance (y-axis) was measured in the square of Person product-moment correlation coefficients (r^2^) of the prediction versus the actual eye position. This figure illustrates that in our dataset the maximum possible performance is r^2^ = 0.87 (N_x,min_ = 0°, N_m_ = 0°). This value decreases with increasing values of the investigated parameters. Further, there is only a small difference between the lines N_x,min_ = 0°, and N_x,min_ = 4°. The line representing N_x,min_ = 8° shows r^2^ values 0.71–0.76. The line representing N_x,min_ = 12° drops even further to r^2^ values of approximately 0.64 with further decrease for small values of N_m_.

The analysis determines the expected level of noise of the saccade integration scheme for the perfect detector of saccades (without false alarms and misses). It also shows that if the detector is capable of reliably estimating magnitudes and directions of saccades between 4–8°, then it can estimate the actual eye gaze angle with the r^2^ more than 0.8.

### Training

The algorithm required three input parameters that had to be calibrated using the ground truth. The training period was defined as the first five minutes of the experiment after stabilization of the HP filter with 0.01 Hz cut-off frequency; the training period lasted approximately 94 seconds. The current detection implementations had significant problems with rejecting false alarms for steeply decreasing/increasing signals. Therefore the initial part of the signal had to be discarded (see Sec. Results). The inputs for training were the EOG signal and the ground truth. In the first step, the saccades were estimated from both the EOG and video-based eye tracker. The EOG saccades were estimated with the current algorithm using a default set of the parameters (f_HP_ = 0.03 Hz, N_x,min_ = 6°, N_m_ = 10°, C_i_ = 700°/mV). In the second step, the ground truth saccades were matched to the EOG based saccades and the parameter C_i_ was estimated using a simple linear regression with one linear parameter. In the third step, the algorithm was run again (f_HP_ = 0.03 Hz and C_i_ set to a new value) for different values of the N_m_, and N_x,min_ parameters in order to find the optimal combination in terms of across-subject mean r^2^ values. The values N_m_ = 9° and N_x.min_ = 8° represent the global maximum for the current dataset. Subsequently, the estimated parameters C_i_, N_m_, and N_x,min_ were used to run the algorithm on the data in the testing period.

## Results

The experiment was designed in order to characterize in-ear EOG measurements and to test whether the saccade integration algorithm using only single-channel recordings could reconstruct horizontal eye gaze angles.

### Raw data

Fig 1A and 1C show two samples of raw in-ear EOG recordings over 22 minutes. Fig 1B and 1D show the details of the EOG waveforms together with the positions of the visual targets. These data illustrate the magnitude of the DC drift in comparison to the magnitude of the EOG signal. Fig 1B demonstrates that the EOG signal correlates with the eye gaze angle and that this relationship can be influenced by DC drift (Fig 1D). Fig 1D shows the case of high DC drift, where it is more difficult to see the relationship between the EOG signal and the ground truth. Transient events that relate to the saccades, however, are still visible in the raw data.

### In-ear EOG

**Fig 7:**
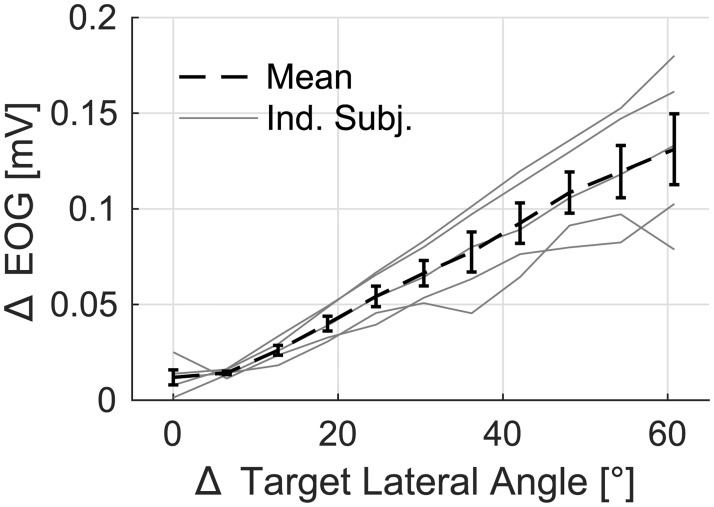
The change in the in-ear EOG as a function of the change of target angle. The magnitude of the change in the visual target is shown on the x-axis. The change was defined as the difference between the medians of the pre-transition and post-transition periods. This was computed for each trial and each participant. Data of individual participants are shown using thin grey lines, the across-subject mean value and SEM error bars are shown with the dashed line.

In order to characterize in-ear EOG and its relationship to eye movements, Fig 7 shows the magnitude of change in the EOG signal as a function of the change of position in the visual target. Each thin grey line shows data from one participant, and the dashed line shows across-subject means. For each transition of the target location, a 300 ms pre-transition to 600 ms post-transition window was used, and the change in the EOG signal was defined as the median value of the first 120 ms minus the median of the last 120 ms of the 900 ms period. The data were pooled across initial target angles and repetitions, and medians were computed for each magnitude of change. This method was preferred over using the linear regression, as in the case of C_i_ computation, in order to avoid the assumptions of the current technique.

Fig 7 illustrates the linear relationship between the change of the target angle and the change of EOG in the range of investigated eccentricities, confirming previous results [16] when EOG was measured in the vicinity of eyes. The data also show that a change of 1° of visual angle corresponded to 2.2 ± 0.5 μV (mean ± 95% CI). The across-subject variance could be a consequence of various factors including the electrode contact, the shape of the head, and the individual differences in the corneo-retinal potential (CRP). The CRP is the source of EOG and it is known to vary with the luminance of the visual scene [11], but it can also be influenced by individual differences in eye physiology.

### Training performance

The performance of the whole algorithm was evaluated in terms of the parameters of a linear model (standard deviation and gain) and the proportion of variance explained by the linear model (r^2^). The saccade detection part of the algorithm was evaluated in terms of F-scores, a measure which takes into account hits, misses, and false alarms [10,15]. The F-score is a measure based on true positive rates (TPR), the percentage of true positives with respect to all true events, and positive predictive values (PPV), the percentage of true positive events with respect to all detected events by this method. The events were saccades obtained from the ground truth measurements greater than N_x,min_. Greater F indicates better detection; an F equal to 1 indicates perfect performance.

$$F=2\times \frac{\left(TRP\times PPV\right)}{\left(TRP+PPV\right)}$$ (10)

The F-score does not take into account the magnitude and direction of the saccade. In terms of our algorithm, the magnitudes of the saccades were important, because we aimed to reconstruct the actual eye gaze angle by integrating saccades. Therefore, this measure was introduced only to allow comparison with the previous work.

**Fig 8:**
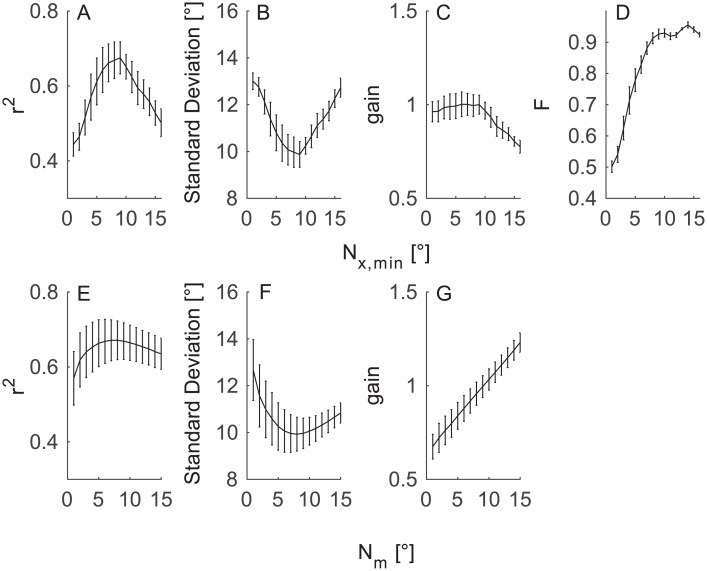
Performance of SACCINT during the training period. The figure shows across-subject mean (±SEM) performance as a function of N_x,min_ parameter (top row) and N_m_ parameter (bottom row). (A, E) The r^2^ values of the actual and predicted eye angles, greater r^2^ indicates better performance. (B, F) the standard deviation of error of the linear model. (C, G) The gain of the linear model. (D) Detection performance in F values (f_HP_ = 0.03 Hz).

During the training period, the performance was evaluated at different values of the N_x,min_ and N_m_ parameters. Fig 8 shows a subset of the training dataset for (Fig 8A–8D) N_m_ = 9° and (Fig 8E–8G) N_x,min_ = 8° while varying the other parameter (f_HP_ was set to 0.03 Hz). N_x,min_ is the parameter of the detection step, thus the panel D shows how the F ratio was influenced by this parameter. N_m_ is the parameter of the integration step; the detection was not influenced by this parameter.

The upper row of Fig 8 shows that r^2^ peaks for the intermediate values of N_x,min_, and it has a maximum of 0.64. The standard deviation of error shows a similar but opposite pattern as r^2^; the smallest value was 10°. The gain of the linear model is constant around a value of 1 for N_x,min_ up to 10° and then decreases. On the other hand, the F-score increases monotonically from ~0.49 at 0° to ~0.93 at 9° and then slowly increases further up to ~0.95 at 14°. The bottom row shows similar patterns for the r^2^ (Fig 8E) and the error statistics (Fig 8F); however, the gain statistic increases monotonically with N_m_.

The upper row illustrates that it is beneficial to restrict the detection algorithm to saccades of certain magnitude by setting the N_x,min_ parameter. The analysis also shows that the N_x,min_ parameter mostly affects the error of the linear model. The bottom row shows that N_m_ influences the slope of the estimated values. For example, the monotonic increase of the gain (Fig 8G) illustrates how the estimated eye positions become more ‘compressed’ with greater N_m_. Gain < 1 indicates that the output values systematically undershoot the true values; gain > 1 indicates overshooting.

### Testing performance

In this section, we compare the SACCINT algorithm against the traditional EOGHP approach during the testing period for various cut-off frequencies. In order to obtain the output values of eye positions, the HP filtered data were multiplied by C_i_/2 and clipped at ±EA_max_ (±35°).

**Fig 9:**
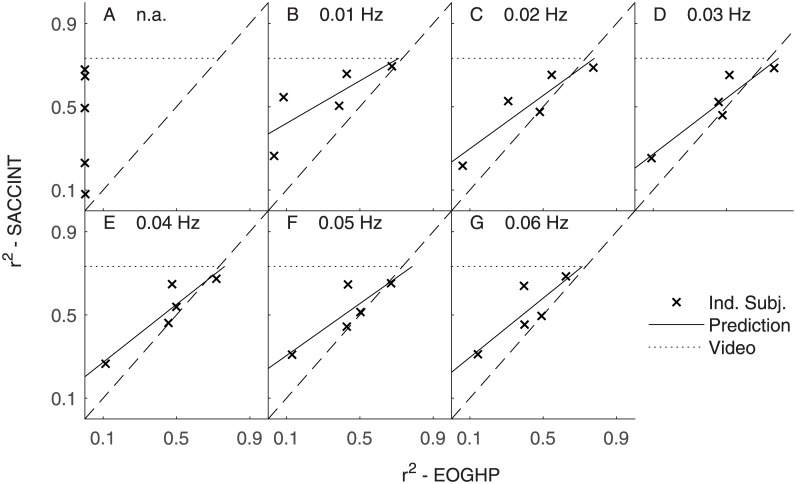
SACCINT vs. EOGHP. Data of individual participants are shown by ‘x’ symbols. The x-axis shows the performance of the EOGHP. Y-axis shows the performance of the SACCINT. Each panel shows data for single cut-off frequency of the HP filter. Solid lines show predictions of the performance of SACCINT obtained from fitting a linear function on the individual data. The dotted line shows the theoretical maximum of the saccade integration for the current set of parameters. The saccades were obtained from the video based eye tracker were delayed by the time of half of the length of the temporal window for the purpose of this comparison and they were integrated with the same parameters as the EOG based data; N_m_ = 9° and N_x,min_ = 8°.

Fig 9 shows the performance of SACCINT for each participant (‘x’ symbol) versus the performance of EOGHP as a function of f_HP_. The figure shows that ‘x’ symbols lie above the dashed lines in the most cases. One participant is below the dashed lines for certain f_HP_, and one other participant is below the dashed line for f_HP_ = 0.03 Hz. This suggests that our novel algorithm SACCINT performs better than a simple HP filter (EOGHP) in most cases. The performance of SACCINT improves with increasing performance of the HP filtering approach.

The results also demonstrate that the SACCINT approach is almost independent of the value of f_HP_. The performance of some participants nearly approached the theoretical limit of saccade integration. The difference between ideal performance (dotted horizontal line) and the performance of the participants (‘x’ symbols) can be attributed only to the quality of the detection step. Fig 9B also shows that the overall performance is limited by the measurements of one particular participant. It is possible that the electrodes had poor contact during this measurement which decreased the quality of the signal (see Sec. Errors in saccade detection).

**Fig 10:**
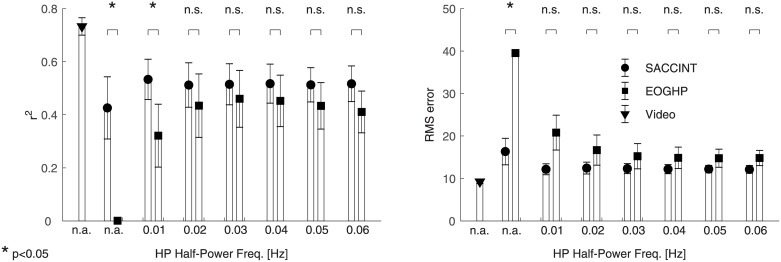
Across-subject performance of SACCINT and EOGHP. (A) The left panel shows across-subject r^2^ values, separately for SACCINT (circles), EOGHP (squares), and the “ideal” performance for the current set of parameters obtained from the ground truth (triangle) using the same parameters. The second and third columns show the data without HP filtering. (B) The right panel shows across-subject RMS error using the same symbols.

The above observations can be summarized in the across-subject analysis of r^2^ (Fig 10A) values and the analysis of RMS errors (Fig 10B). These data show that the saccade integration scheme achieved maximum performance of r^2^ = 0.54 ± 0.14 (95% CI) for f_HP_ = 0.01 Hz, and the performance changed slightly with varying HP cut-off frequency. The across-subject performance of the EOGHP was poor (r^2^ = 0) when no HP filter was applied. The performance improved with increasing cut-off frequency and peaked at r^2^ = 0.46 for f_HP_ = 0.03 Hz. It then decreased for greater cut-off frequencies. The SACCINT performed better than the EOGHP for some f_HP_ but not all. SACCINT performed significantly better than EOGHP without the HP filter (two-tailed paired t-test, t_4_ = 3.6244; p < 0.05, controlled for false discovery rate [35]) and with f_HP_ = 0.01 Hz (t_4_ = 2.8886; p < 0.05).

The RMS error of the SACCINT had minimum 12° ± 2° (95% CI) for f_HP_ = 0.01 Hz. The performance was approximately constant for the set of investigated frequencies. The RMS error of the EOGHP was 15° for f_HP_ = 0.03 Hz and above, and this value was elevated for decreasing the cut-off frequency. The only statistically significant difference between SACCINCT and EOGHP was without HP filtering (t_4_ = 8.8034; p < 0.05).

These data illustrate that even EOGHP could predict eye gaze angle from in-ear EOG measurements. The SACCINT algorithm is a more robust way of estimating the eye gaze angle than the standard HP filtering. The performance of the EOGHP approach is strongly influenced by the f_HP_ value. The fact that EOGHP was not significantly different from SACCINT for most f_HP_ values partly relates to the design of the experiment. For example, fixation periods > 1 s would deteriorate the EOGHP approach, but would not affect the new saccade integration scheme SACCINT.

### Errors in saccade detection

**Fig 11:**
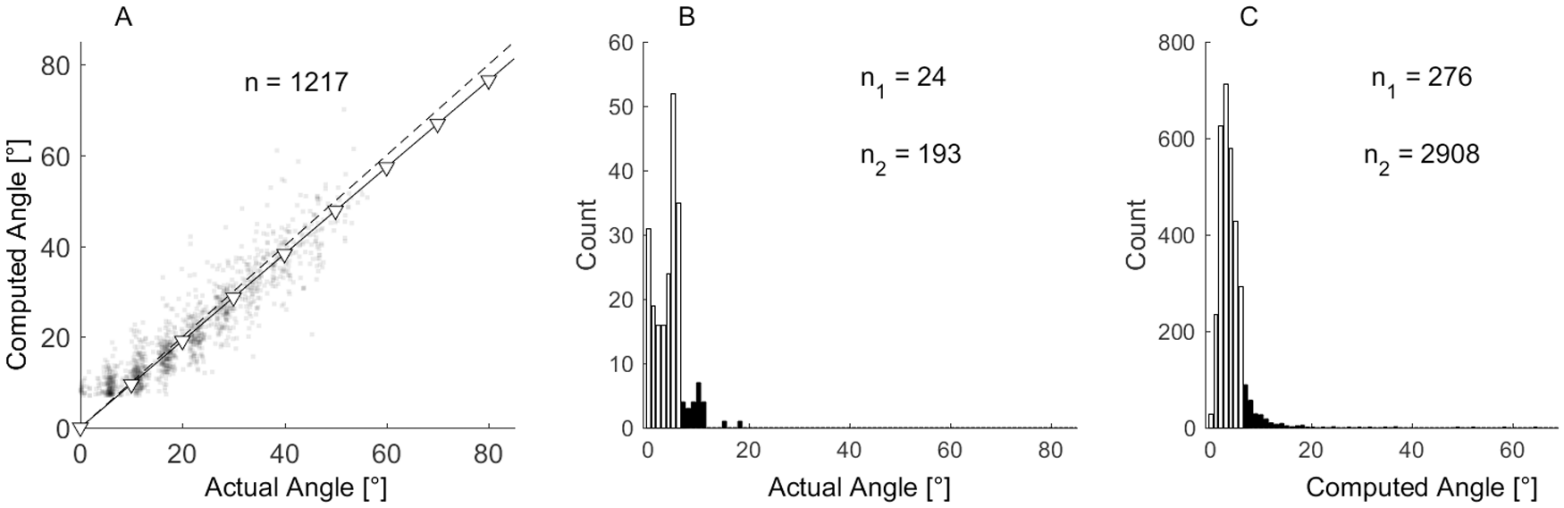
Saccade detection evaluation for one participant. (A) Scatter plot of the computed versus the actual saccade magnitudes. The solid line with open triangles shows the linear regression with the constant term fixed to zero. (B) Histogram of missed saccades, the white bars show misses smaller than N_x,min_ (8°), the black bars show misses larger than N_x,min_. (C) Histogram of false alarms. White bars in the histogram show the data of saccades with magnitude smaller than N_x,min_ (8°), black bars show the rest of the dataset. The numbers inside panels indicate a total number of points in each histogram. Subscript 1 refers to the black bars, and subscript 2 refers to the white bars.

In order to analyse the detection algorithm of SACCINT, Fig 11 shows the patterns of errors of a representative participant during the testing phase (f_HP_ = 0.03 Hz). Panel Fig 11A shows that computed saccades only slightly underestimated the magnitude of the actual saccades. When the slope was fitted to the data of all participants, it had a value of 0.95 ± 0.05 (mean ± 95% CI). This deviation from 1 relates to the selection of N_m_ = 9° in the training phase and the cut-off frequency of the HP filter. The standard deviation of the error between the fit and the matched saccades was 4.67°± 0.9 (across-subject mean ± 95% CI), which characterizes high-frequency noise inherently present in the EOG signal and the precision of the saccade magnitude estimation. Panel Fig 11B shows the relatively small number of misses and relatively large number of undetectable saccades, which suggests that the improvements in the saccade detection algorithm may improve the performance. Fig 11C shows that the majority of the correct rejections were of small magnitudes and they were filtered out at the detection step. Notably, the algorithm still reports a substantial number of false alarms (276) which is 22% of all real saccades.

**Fig 12:**
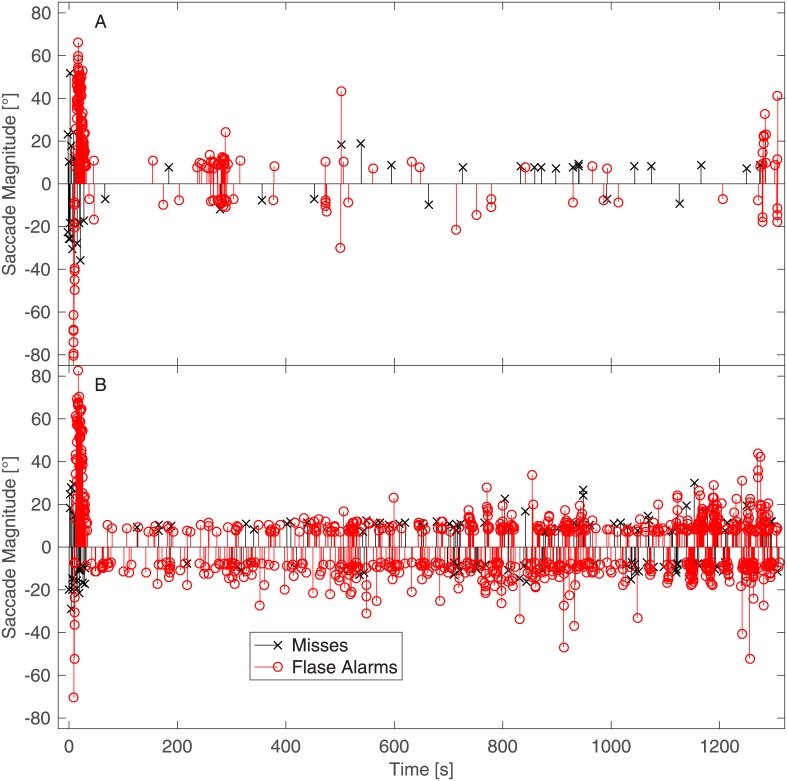
Temporal distribution of missed and falsely alarmed saccades of two example participants.

Fig 12 provides further insight into the patterns of errors of the saccade detection algorithm. The figure shows the temporal distributions of the misses and false alarms of the two example participants whose raw EOG traces are shown in Fig 1. When Fig 12 is compared with Fig 1 (Fig 12A corresponds to Fig 1A and 1B and Fig 12B corresponds to Fig 1C and 1D), three characteristics of the algorithm’s errors are shown. First, the number of false alarms dramatically increased if the signal had a very strong DC component (Fig 1C) and contained more high-frequency noise (Fig 1D). That is, the areas of densely distributed false alarms being in one direction indicate that they had a common origin from the DC component; the false alarms in both directions were present due to high-frequency noise. Second, the number of misses was approximately constant. Third, many false alarms were due to the sloping signal before the DC filter stabilised.

Taken all together, this indicates that the high false alarm rate was the most prominent constraint of the current implementation. A proper statistical model of the eye movements (e.g., [36]) should eliminate the majority of the observed errors.

## Discussion

The experiment and analysis demonstrated that it is possible to estimate horizontal eye gaze angle using a single-channel EOG measurement with a pair of ear moulds positioned inside the ear canal. However, the estimates are still noisy. SACCINT achieved the across-subject performance of r^2^ = 0.54, which was better than a simple EOGHP approach for some values of f_HP_. The performance of the EOGHP depends on the fixation period and the f_HP_, while SACCINT is independent of the fixation period and much less dependent on f_HP_. Therefore, it is likely that SACCINT would outperform the EOGHP in scenarios involving movements and real visual targets once the saccade detection step is improved. However, only five participants were tested in this study in a very controlled environment (e.g., with heads fixed), and with custom-made electrodes. Therefore, the current results have to be understood in the context of these and other limitations.

Firstly, the quality of the EOG measurements is critical for any EOG-based technology, and it was observed that the quality varied across participants. This can be related to the quality of our custom made electrodes, and it is very likely that better electrodes can substantially improve this system.

Secondly, the analysis of errors showed that most of the errors were introduced by the detection algorithm. Many of the errors could be eliminated by more advanced methods of detection; for example, by probabilistic classification [15]. If the detection was ideal, the performance could achieve an r^2^ of 0.8. One of the problems was that the current model could not properly describe the drifting EOG signal (e.g., when the EOG was dominated by the DC drift, or when the HP filter was introduced), because this type of signal was always incorrectly interpreted as a series of saccades in one direction. This resulted in a large number of false alarms. This particular problem limits the effectiveness of the algorithm. The number of false alarms can be reduced in the future by modifying the model such that it captures the drift more closely. The detection can be further improved by employing a more advanced model of eye physiology [36] that, for example, assumes refectory periods between saccades and natural rates of saccade occurrence, which can reduce the rate of false alarms. The statistics of eye movements [37] and statistics of eye movements in relation to the eye position [38,39] could also be implemented to improve the detection step. However, EOG does not contain any information about the environment or the intention of the participant (e.g., whether the saccade is voluntary or involuntary). Therefore any statistical model would have to take into account only eye physiology, natural tendencies of the eye movements, or factors which are not dependent on environment or task.

Thirdly, the integration step was a simplistic model of the eye physiology assuming a perfectly constant eye position during the fixation and a step-like change of eye position during a saccade. This introduced noise into the estimation process because the eye is never perfectly still. Further, the additional noise was introduced because the estimated saccades were delayed by approximately half of the duration of the window length. Implementing a statistical predictor of the eye position, however, could improve the integration step. A possible future predictor of the eye position is the head angle. The contributions of head movements to the eye gaze directions have been previously demonstrated, for instance, in a visual search task experiment [34] with stimuli distributed over 360° around the participant with unconstrained head movements. That experiment showed that the distributions of the eye gaze directions followed the distributions of the head vs. body orientation (e.g., when the head turns to left, the eye gaze is likely to be to the left). That means that if the saccade integration model had access to the actual head vs. body orientation, then it has an independent predictor of eye position. However, the head was fixed in the current experiment, and the positions of the visual targets were strictly controlled. Future experiments should, therefore, test the method in more realistic environments that include head movements.

### Relation to previous work

#### In-ear EOG

The magnitude of the in-ear EOG signal was estimated to be 2.2 μV per 1° of visual angle, which is less than the measurements in the standard position and slightly less than the previously reported in-ear measurement of 3 μV /1° [18]. One of the five participants had an in-ear EOG magnitude of 2.8 μV/1° which is closer to the previous study. The relationship of the change in EOG to the change in target angle was linear for the observed transition magnitudes up to 61°. In our experiment, the saccade transitions were not uniformly distributed, and the eye positions with eccentricities larger than 30.5° were not tested. Therefore, further testing is necessary to establish whether linearity is preserved for extreme eye positions.

#### Saccade detection and saccade identification

Previously, the saccades were detected by analysing parameters of the continuous wavelet transformation [10]; the detector performed with small number of errors (F = 0.94). In that study, they detected the magnitudes of the saccades, but these were only classified as either small or large, and the actual eye angle was not estimated. Barea et al. [16] reported that their system was able to identify saccades with magnitudes greater than 10° and an error of 2°. A method of Iáñez et al. [14] was based on an analysis of the derivative of EOG. Detection performance was the same as [11] (F = 0.94). Another method based on continuous wavelet transformation and auto-calibration [21] claimed almost perfect detection of horizontal saccades, but the analysis was based on an offline artifact and drift removal step. When the output was compared to the eye tracker data, the performance diminished. Vidal et al. [20] based their method on feature extraction (velocity, acceleration, slope, parameters of polynomial fit) and also claimed almost perfect detection. The study did not report the length of the window that was used for the data analysis. Therefore, it was not clear whether the data were processed offline or online. These various methods usually achieved similar or better performance than our algorithm, but in all these studies, the EOG signal was measured at the peri-orbital positions which offer a substantially higher signal to noise ratio. The studies also tended to use signals from two or more channels (e.g., horizontal and vertical channels), and they disregarded the magnitudes of the saccades when evaluating the performance of the detection algorithm (e.g., a small saccade could be assigned as a large saccade). Thus, it is not possible to directly compare the current results with the performance of the mentioned methods.

#### Eye angle estimation

Several previous studies [18,22,23,40,41] have suggested the possibility of estimating eye gaze angle from EOG. These methods either relied on readings from multiple electrodes, or they used a camera for calibration. One approach [22] analysed the EOG signal from multiple electrodes, which allowed to decorrelate the noise components in different electrodes while preserving the EOG signal, thus allowing direct estimation of gaze angle. In another approach [23], gaze angle estimation was based on the EOG signal that was calibrated by saliency maps obtained from an external camera. The estimation RMS error of the current method was 12° which is greater than the multiple-electrode method [22] error of 4°, but less than the externally calibrated method [23] error of 15°. However, the current and the previous methods cannot be compared directly because neither of the previous experiments measured the EOG signal inside the ear canals with just two electrodes.

### Hearing aids and other applications

The motivation for the current investigation was the development of a portable and unobtrusive eye gaze angle estimation technique for hearing aids. This could be used to steer directional microphones toward attended sounds [4]. This technology could engage the hearing-impaired listeners into dynamic conversations in noisy environments, and make such situations less challenging because the listener usually consciously looks at the attended talker(s). However, eye movements (as well as head movements) reflect exogenous attention which might have a detrimental effect on listening, if the acoustic beam of the directional microphone system was too narrow [42]. Therefore, future research is needed to estimate the effect of the parameters of such technology on their benefit in real listening scenarios.

Potentially, eye-movement information could provide information about the health, mood, or environment where the user currently is. For instance, previous research studied eye movements in connection with various types of neurodegenerative disease [43]. Further, this type of eye tracking could be incorporated in consumer headphones, virtual reality systems, or systems that monitor fatigue. Portable eye tracking techniques have been previously used in marketing [44], sport [45], sleep [13], and car driving [46] research. Less intrusive methods of eye tracking could ease the data collection in realistic environments.

### Limitations

The most notable limitation of all EOG applications is the signal to noise ratio, which relates mainly to measurement artifacts: DC drift and muscular activity. The application is further constrained by the assumptions of the current model, specifically its saccade detection and integration. The algorithm is only capable of detecting (with relative reliability) saccades of large magnitude (> 8°). The magnitude estimation, though, is accurate; mean estimation error was 4.67°. The algorithm cannot detect smooth pursuit nor any other smooth eye movement. Smooth pursuit and DC drift appear identical in the EOG signal, which means that they are impossible to distinguish in an online analysis. Other limitations are the parameters of the saccade integration model. In this work, these were estimated in the training period but in real environments, they may change over time, and they need to be calibrated. The parameter C_i_ may change with external lighting conditions. One possible solution is to implement a light sensor and define the relationship between the change of lighting and in-ear EOG. However, that would require an investigation of whether lighting is the only factor that affects the EOG magnitude. A second way to calibrate C_i_ is to use the vestibulo-ocular reflex in connection with head movements. When the head moves, eyes often remain fixed during head movements, thus if the device was equipped with a gyroscope, then the strongly correlated outputs of the EOG and gyroscope can instantaneously calibrate the system. Two other parameters N_m_ and N_x,min_ are specific for this particular implementation, which means that they might need to be replaced in the future. Moreover, N_m_ and N_x,min_ are not likely to change over time.

Another limitation of the current approach is that our tests were conducted only in one controlled environment, with a small sample of participants, without deliberate body movements, and with targets uniformly spaced across the visual field. Nevertheless, several previous studies have demonstrated that EOG can be measured even in a mobile environment [9,10,23], and showed that specialized algorithms can diminish the effects of commonly occurring artifacts (e.g., the walking artifact). Certain artifacts cannot be filtered easily. For example, the artifacts related to the jaw or tongue movements would be difficult to filter, because they do not have any regular shape or frequency, and any EOG-to-gaze algorithm is vulnerable to them.

The current algorithm can be used online, but this analysis was run offline on a PC with high computational power. In this implementation, the most computationally demanding step is the non-linear fitting procedure, but a different fitting procedure may be more computationally efficient. One further limitation is that the current approach analysed the signal in a temporal window, which in practice would lead to a delay up to 200 ms, and this delay cannot be avoided.

## Conclusion

The current work showed that it is possible to estimate the eye gaze angle with a single-channel in-ear EOG recording using EOGHP (r^2^ = 0.46) and a novel SACCINT (r^2^ = 0.54) method. The estimates were still noisy, but in theory the SACCINT could attain much better performance (r^2^ > 0.8). This difference between the theory and the actual performance of the SACCINT can be attributed mostly to the quality of the in-ear EOG signal, which lead to errors in the detection and integration steps. Therefore, further improvements of this method are necessary. A number of improvements have been proposed, including improving the design of the electrodes, improving the non-linear fitting procedure, modelling of eye physiology, incorporating gyroscope signals, or incorporating statistical models. Overall, our investigation suggests that in-ear EOG signals captured with conductive ear moulds could serve as a basis for light-weight, portable horizontal eye gaze angle estimation suitable for broad range of applications not limited to hearing aids.

## References

[1] Grant KW, Walden BE, Seitz PF. Auditory-visual speech recognition by hearing-impaired subjects: Consonant recognition, sentence recognition, and auditory-visual integration. J Acoust Soc Am. 1998;103: 2677–2690. doi: 10.1121/1.422788 9604361 10.1121/1.422788

[2] Bernstein LE, Demorest ME, Tucker PE. Speech perception without hearing. Percept Psychophys. 2000;62: 233–252. doi: 10.3758/BF03205546 10723205 10.3758/bf03205546

[3] Desai S, Stickney G, Zeng F-G. Auditory-visual speech perception in normal-hearing and cochlear-implant listeners. J Acoust Soc Am. 2008;123: 428–440. doi: 10.1121/1.2816573 18177171 10.1121/1.2816573 PMC2662523

[4] Kidd G Jr., Favrot S, Desloge JG, Streeter TM, Mason CR. Design and preliminary testing of a visually guided hearing aid. J Acoust Soc Am. Boston, MA, United States.: Acoustical Society of America; 2013;133: EL202–EL207. doi: 10.1121/1.4791710 23464129 10.1121/1.4791710 PMC3585754

[5] Hart J, Onceanu D, Sohn C, Wightman D, Vertegaal R. The Attentive Hearing Aid: Eye Selection of Auditory Sources for Hearing Impaired Users. Lecture Notes in Computer Science (including subseries Lecture Notes in Artificial Intelligence and Lecture Notes in Bioinformatics). 2009. pp. 19–35. 10.1007/978-3-642-03655-2_4

[6] Bulling A, Kunze K. Eyewear computers for human-computer interaction. interactions. 2016;23: 70–73. doi: 10.1145/2912886

[7] Ramli R, Arof H, Ibrahim F, Mokhtar N, Idris MYI. Using finite state machine and a hybrid of EEG signal and EOG artifacts for an asynchronous wheelchair navigation. Expert Syst Appl. Elsevier Ltd; 2015;42: 2451–2463. doi: 10.1016/j.eswa.2014.10.052

[8] Barea R, Boquete L, Mazo M, López E. Wheelchair guidance strategies using EOG. J Intell Robot Syst Theory Appl. 2002;34: 279–299. doi: 10.1023/A:1016359503796

[9] Bulling A, Roggen D, Tröster G. Wearable EOG goggles: Seamless sensing and context-awareness in everyday environments. J Ambient Intell Smart Environ. 2009;1: 157–171. doi: 10.3233/AIS-2009-0020

[10] Bulling A, Member S, Ward JA, Gellersen H, Tro G, Tröster G. Eye movement analysis for activity recognition using electrooculography. IEEE Trans Pattern Anal Mach Intell. 2011;33: 741–753. doi: 10.1109/TPAMI.2010.86 20421675 10.1109/TPAMI.2010.86

[11] Brown M, Marmor M, Vaegan, Zrenner E, Brigell M, Bach M. ISCEV Standard for Clinical Electro-oculography (EOG) 2006. Doc Ophthalmol. 2006;113: 205–212. doi: 10.1007/s10633-006-9030-0 17109157 10.1007/s10633-006-9030-0 PMC1820752

[12] Haslwanter T, Clarke AH. Eye movement measurement. electro-oculography and video-oculography [Internet]. 1st ed Handbook of Clinical Neurophysiology. Elsevier B.V.; 2010 doi: 10.1016/S1567-4231(10)09005-2

[13] McPartland RJ, Kupfer DJ. Computerised measures of electro-oculographic activity during sleep. Int J Biomed Comput. 1978;9: 409–419. doi: 10.1016/0020-7101(78)90048-X 216636 10.1016/0020-7101(78)90048-x

[14] Iáñez E, Azorin JM, Perez-Vidal C. Using Eye Movement to Control a Computer: A Design for a Lightweight Electro-Oculogram Electrode Array and Computer Interface. PLoS One. 2013;8: 1–10. doi: 10.1371/journal.pone.0067099 23843986 10.1371/journal.pone.0067099 PMC3700965

[15] Toivanen M, Pettersson K, Lukander K. A probabilistic real-time algorithm for detecting blinks, saccades, and fixations from EOG data. J Eye Mov Res. 2015;8: 1–14. doi: 10.16910/jemr.8.2.1

[16] Barea R, Boquete L, Ortega S, López E, Rodríguez-Ascariz JM. EOG-based eye movements codification for human computer interaction. Expert Syst Appl. Elsevier Ltd; 2012;39: 2677–2683. doi: 10.1016/j.eswa.2011.08.123

[17] Puthusserypady S, Ratnarajah T. Robust adaptive techniques for minimization of EOG artefacts from EEG signals. Signal Processing. 2006;86: 2351–2363. doi: 10.1016/j.sigpro.2005.10.018

[18] Manabe H, Fukumoto M. Using Earphones to Perform Gaze Detection for Warable Interfaces. NTT DOCOMO Tech J. 2006;12: 12–17.

[19] Behrens F, MacKeben M, Schröder-Preikschat W. An improved algorithm for automatic detection of saccades in eye movement data and for calculating saccade parameters. Behav Res Methods. 2010;42: 701–708. doi: 10.3758/BRM.42.3.701 20805592 10.3758/BRM.42.3.701

[20] Vidal M, Bulling A, Gellersen H. Analysing EOG signal features for the discrimination of eye movements with wearable devices. Proceedings of the 1st international workshop on Pervasive eye tracking & mobile eye-based interaction—PETMEI ‘11. New York, New York, USA: ACM Press; 2011. p. 15. 10.1145/2029956.2029962

[21] Pettersson K, Jagadeesan S, Lukander K, Henelius A, Haeggström E, Müller K, et al Algorithm for automatic analysis of electro-oculographic data. Biomed Eng Online. 2013;12 doi: 10.1186/1475-925X-12-110 24160372 10.1186/1475-925X-12-110 PMC3830504

[22] Manabe H, Fukumoto M, Yagi T. Direct Gaze Estimation Based on Nonlinearity of EOG. IEEE Trans Biomed Eng. 2015;62: 1553–1562. doi: 10.1109/TBME.2015.2394409 25615905 10.1109/TBME.2015.2394409

[23] Sugano Y, Bulling A. Self-Calibrating Head-Mounted Eye Trackers Using Egocentric Visual Saliency. Proceedings of the 28th Annual ACM Symposium on User Interface Software & Technology. 2015. pp. 363–372. 10.1145/2807442.2807445

[24] Borji A, Itti L. State-of-the-Art in Visual Attention Modeling. IEEE Trans Pattern Anal Mach Intell. 2013;35: 185–207. doi: 10.1109/TPAMI.2012.89 22487985 10.1109/TPAMI.2012.89

[25] Grimnes S, Martinsen ØG. Bioimpedance and Bioelectricity Basics. Academic Press; 2015.

[26] Nakashima R, Fang Y, Hatori Y, Hiratani A, Matsumiya K, Kuriki I, et al Saliency-based gaze prediction based on head direction. Vision Res. Elsevier Ltd; 2015;117: 59–66. doi: 10.1016/j.visres.2015.10.001 26475088 10.1016/j.visres.2015.10.001

[27] de Bruijn NG. A combinatorial problem. Proc Sect Sci K Ned Akad van Wet te Amsterdam. 1946;49: 758–764.

[28] Kassner M, Patera W, Bulling A. Pupil: An Open Source Platform for Pervasive Eye Tracking and Mobile Gaze-based Interaction. 2014; http://arxiv.org/abs/1405.0006

[29] Kleiner M, Brainard D, Pelli D, Ingling A, Murray R, Broussard C. What’s new in psychtoolbox-3. Perception. 2007;36: 1–16.

[30] Brainard DH. The Psychophysics Toolbox. Spat Vis. 1997;10: 433–436. doi: 10.1163/156856897X00357 9176952

[31] Pelli DG. The VideoToolbox software for visual psychophysics: transforming numbers into movies. Spat Vis. 1997;10: 437–442. doi: 10.1163/156856897X00366 9176953

[32] Krassanakis V, Filippakopoulou V, Nakos B. EyeMMV toolbox: An eye movement post-analysis tool based on a two-step spatial dispersion threshold for fixation identification. J Eye Mov Res. 2014;7(1): 1–10. Available: http://users.ntua.gr/bnakos/Data/Section5-7/Pub_5-7-19.pdf

[33] Zhu C, Byrd RH, Lu P, Nocedal J. Algorithm 778: L-BFGS-B: Fortran subroutines for large-scale bound-constrained optimization. ACM Trans Math Softw. 1997;23: 550–560. doi: 10.1145/279232.279236

[34] Fang Y, Nakashima R, Matsumiya K, Kuriki I, Shioiri S. Eye-head coordination for visual cognitive processing. PLoS One. 2015;10: 1–17. doi: 10.1371/journal.pone.0121035 25799510 10.1371/journal.pone.0121035 PMC4370616

[35] Glickman ME, Rao SR, Schultz MR. False discovery rate control is a recommended alternative to Bonferroni-type adjustments in health studies. J Clin Epidemiol. 2014;67: 850–857. doi: 10.1016/j.jclinepi.2014.03.012 24831050 10.1016/j.jclinepi.2014.03.012

[36] Komogortsev O V, Khan JI. Eye Movement Prediction by Kalman Filter with Integrated Linear Horizontal Oculomotor Plant Mechanical Model. Eye Track Res Appl Symp. 2008; 229–236. doi: 10.1145/1344471.1344525

[37] Boccignone G. Advanced statistical methods for eye movement analysis and modeling: a gentle introduction. 2015; http://arxiv.org/abs/1506.07194

[38] Tatler BW, Vincent BT. The prominence of behavioural biases in eye guidance. Vis cogn. 2009;17: 1029–1054. doi: 10.1080/13506280902764539

[39] Le Meur O, Coutrot A. Introducing context-dependent and spatially-variant viewing biases in saccadic models. Vision Res. Elsevier Ltd; 2016;121: 72–84. doi: 10.1016/j.visres.2016.01.005 26898752 10.1016/j.visres.2016.01.005

[40] Manabe H, Fukumoto M. Full-time Wearable Headphone-Type Gaze Detector. 2006; 1073–1078.

[41] Manabe H, Fukumoto M, Yagi T. Automatic drift calibration for EOG-based gaze input interface. Proc Annu Int Conf IEEE Eng Med Biol Soc EMBS. 2013; 53–56. 10.1109/EMBC.2013.6609435 24109622

[42] Hládek Ľ, Porr B, Brimijoin WO. Effect of width of acoustic beam in eye-controlled beamforming in a dynamic “cocktail party.” BSA Basic Auditory Science, University of Nottingham, September 4–5. 2017.

[43] Anderson TJ, MacAskill MR. Eye movements in patients with neurodegenerative disorders. Nat Rev Neurol. Nature Publishing Group; 2013;9: 74–85. doi: 10.1038/nrneurol.2012.273 23338283 10.1038/nrneurol.2012.273

[44] Higgins E, Leinenger M, Rayner K. Eye movements when viewing advertisements. Front Psychol. 2014;5: 1–15. 24672500 10.3389/fpsyg.2014.00210 PMC3956003

[45] Shank MD, Haywood KM. (University of Missouri-St. Louis) Eye movements while viewing a baseball pitch1. Percept Mot Skills. 1987; 1191–1197. doi: 10.2466/pms.1987.64.3c.1191

[46] Schleicher R, Galley N, Briest S, Galley L. Blinks and saccades as indicators of fatigue in sleepiness warnings: Looking tired? Ergonomics. 2008;51: 982–1010. doi: 10.1080/00140130701817062 18568959 10.1080/00140130701817062

